# Two case reports of small cell lung cancer: typical CT manifestations, with one case illustrating adjunctive CT-based diagnosis without pathological confirmation

**DOI:** 10.3389/fonc.2026.1865534

**Published:** 2026-10-21

**Authors:** Yibing Zhu, Qingyu Wang, Meiqi Yu, Yinggang Qin, Li Sun, Ming Ye, Junyi Li, Huibin Huang

**Affiliations:** 1 Department of Emergency, Guang’anmen Hospital, China Academy of Chinese Medical Sciences, Beijing, China; 2 Department of Oncology, Guang’anmen Hospital, China Academy of Chinese Medical Sciences, Beijing, China; 3 Department of Orthopedics, Guang’anmen Hospital, China Academy of Chinese Medical Sciences, Beijing, China; 4 Department of Radiology, Guang’anmen Hospital, China Academy of Chinese Medical Sciences, Beijing, China; 5 Department of Nephrology, Guang’anmen Hospital, China Academy of Chinese Medical Sciences, Beijing, China; 6 Department of Surgery, Guang’anmen Hospital, China Academy of Chinese Medical Sciences, Beijing, China

**Keywords:** case report, computed tomography, diagnosis, imaging, small cell lung cancer

## Abstract

**Background:**

Small cell lung cancer (SCLC) is a highly malignant neuroendocrine tumor with rapid progression and poor prognosis. Pathological examination is the gold standard for its diagnosis, yet pathological sampling is unfeasible in some clinical scenarios due to various constraints, making imaging and tumor markers critical for clinical decision-making. This two-case report describes the typical computed tomography (CT) findings of SCLC and explores the clinical value of combining CT imaging with tumor markers in guiding the diagnosis and treatment of SCLC patients for whom pathological confirmation is not feasible.

**Case report:**

We report two SCLC cases: Case 1 was a 56-year-old male with pathologically confirmed SCLC who presented typical imaging findings of hilar and mediastinal fused lymph nodes and achieved a partial response after combined chemoradiotherapy. Case 2 was a 67-year-old male with chest tightness and hemoptysis for 2 months, whose chest CT showed fused lymph nodes (a typical SCLC feature) and markedly elevated neuron-specific enolase (NSE: 221 ng/mL; reference range 1-16.2 ng/mL). Biopsy was unfeasible due to recent coronary stenting and ongoing antiplatelet therapy, and a clinical diagnosis of limited-stage SCLC was made via a combination of typical CT imaging features, significantly elevated NSE and clinical symptoms. The patient received timely EC regimen chemotherapy and subsequent standard chemoradiotherapy, which resulted in a clinical complete response (CR).

**Conclusions:**

This case report details the typical CT manifestations of SCLC and demonstrates that the combined application of typical CT findings, tumor markers and clinical symptoms may provide a pragmatic basis for timely clinical diagnosis and treatment of SCLC patients when pathological sampling is not achievable. This approach may help avoid treatment delay and improve clinical outcomes for such patients, while emphasizing that pathological examination remains the gold standard for SCLC diagnosis.

## Introduction

Small cell lung cancer (SCLC) is a highly aggressive neuroendocrine tumor that accounts for approximately 13%–15% of all lung cancers (1–3). It progresses rapidly, metastasizes early, and carries a 5-year survival rate of only 5%–10% (4–6); most patients already have extensive-stage disease at diagnosis. Timely diagnosis and treatment initiation are therefore critical to improving outcomes.

Pathological examination remains the diagnostic gold standard (7), but tissue sampling is not always feasible. Patients with recent coronary stent implantation require uninterrupted antiplatelet therapy, and invasive biopsy either carries a high bleeding risk or necessitates a 5–7-day washout that itself increases thrombotic risk (8). Poor general condition or inaccessible tumor location may further preclude sampling. Such delays are particularly hazardous in SCLC, whose tumor doubling time is only 25–30 days (7): a few weeks’ delay may allow progression from limited-stage to extensive-stage disease. In these scenarios, chest CT becomes central to evaluation. SCLC characteristically presents as a central mass with early, coalescent hilar and mediastinal lymphadenopathy (9–11)—an imaging pattern that helps distinguish it from other thoracic diseases.

Current guidelines, including those of the NCCN, mandate pathological confirmation and provide no dedicated guidance for diagnosis when tissue cannot be obtained (7, 12), and published evidence addressing this scenario is scarce. Here we report two SCLC cases with typical CT features—one pathologically confirmed, the other clinically diagnosed when biopsy was contraindicated—to illustrate how characteristic imaging, combined with tumor markers and clinical presentation, can support timely diagnosis and treatment when pathology is unobtainable.

## Case report

### Case 1

A 56-year-old male patient presented to the Department of Oncology of our hospital with complaints of “facial and head edema, and cough with blood-tinged sputum for 2 months.” The patient had a past medical history of eczema for 2 years, with no other relevant medical conditions. He had a smoking history of more than 10 years, with an average of approximately 20 cigarettes per day, and had quit smoking for over 2 years. Regarding family cancer history, there was no documented malignant tumor among his first-degree relatives. The patient lived with his spouse. The patient’s Eastern Cooperative Oncology Group (ECOG) performance status was assessed as 1. Two months prior to admission, the patient developed unexplained facial and head edema without accompanying cough, expectoration, or chest discomfort. One month before admission, he underwent a chest enhanced CT and bronchoscopic biopsy at another hospital, which confirmed small cell neuroendocrine carcinoma (grade 3). Ten days before admission, he completed a PET-CT, which showed right lung cancer with multiple lymph node metastases. On admission, physical examination revealed obvious abnormal dilation of superficial thoracic wall veins. Respiratory examination showed symmetric thoracic expansion, coarse breath sounds in the right upper lung and mild rhonchi over the right middle and lower lung lobes, without pleural friction rub. Cervical, supraclavicular and axillary lymph node palpation detected no palpable enlarged lymph nodes. Cardiovascular examination was unremarkable with regular heart rhythm and normal heart sounds, no murmurs or pericardial friction rubs. Facial and bilateral upper limb pitting edema was observed, consistent with clinical manifestations of superior vena cava syndrome (SVCS). No jaundice, rash or other abnormal skin lesions were noted; abdominal palpation yielded soft abdomen without tenderness or organomegaly, and neurological signs were entirely normal. Since the patient’s pre-hospital diagnosis was clear, and based on the patient’s existing examination results, contraindications were excluded. Three days after admission, the patient received the first cycle of Etoposide + Carboplatin (EC) regimen chemotherapy, specifically: Etoposide injection 100mg on days 1-5 + Carboplatin for Injection 400mg day 1. Images were reviewed independently by two radiologists. This CT scan shows that the findings in the mediastinum, right hilum, and right lung are consistent with small cell lung cancer with multiple metastases. The superior vena cava is involved, with possible involvement of the brachiocephalic vein and right pulmonary vein. There are filling defects in the left subclavian vein and common carotid vein, which may be tumor thrombi. There is an obstructive lesion in the right upper lung lobe and inflammation in the right middle and lower lung lobes. There are a small amount of left pleural effusion and a small amount of pericardial effusion. There is multiple tortuous and thickened vascular shadows under the chest and abdominal wall skin, suggesting possible superior vena cava obstruction syndrome. Four weeks after the first cycle, the second cycle of EC chemotherapy was administered, specifically: Etoposide injection 100mg days 1-5 + Carboplatin for Injection 450mg day1. The carboplatin dose was increased from 400 mg to 450 mg intentionally: the patient’s estimated glomerular filtration rate (eGFR) improved before Cycle 2 without severe hematological toxicity after Cycle 1, and the dose was recalculated via the Calvert formula (13). No related adverse toxicities occurred after dose adjustment. To further eliminate residual tumor cells through precise radiotherapy and reduce the local recurrence rate, the patient subsequently underwent proton radiotherapy at another hospital. After treatment, the symptoms of facial and head edema and superficial vascular dilation were significantly relieved. Six weeks after the second cycle (following proton radiotherapy at another hospital), a follow-up enhanced chest CT showed a significant reduction in the tumor (**Figure 1**; right panel). Compared with the prior imaging, the multiple space-occupying lesions in the mediastinum, right hilum, and right lung have significantly regressed. Metastases to the mediastinal and right hilar lymph nodes have significantly improved compared with the previous. The right clavicular lymph node metastasis seen in the original film is not observed in this examination. The invasion of the superior vena cava, azygos vein, brachiocephalic vein, and right pulmonary arteriovenous in the original film has significantly improved; in this examination, only the superior vena cava and azygos vein are invaded. The tumor thrombi in the left subclavian vein and common carotid vein in the original film are not observed in this examination. The obstructive lesion in the right upper lung lobe, right pleural and interlobar effusion in the original film are not observed in this examination. The small amount of left pleural effusion and small amount of pericardial effusion in the original film are not observed in this examination. The multiple tortuous and thickened vascular shadows under the chest and abdominal wall skin in the original film are not observed in this examination. One week after the follow-up CT, the third cycle of chemotherapy was administered, with the fourth cycle completed four weeks later (efficacy evaluated as partial response [PR]). The patient was staged as an extensive-stage SCLC with cT4N3M1a (stage IVA) according to the AJCC TNM staging system. Prophylactic cranial irradiation (PCI) was considered by the treating team after the patient’s PR to chemoradiotherapy. The patient declined PCI and agreed to regular brain MRI surveillance. Approximately 8 months after the initial treatment, the patient developed dizziness and headache, and an enhanced head MRI revealed a right occipital lobe metastatic tumor, which was treated with cranial volumetric arc radiotherapy. Two years after the initial diagnosis, the patient restarted chemotherapy combined with immunotherapy. As of July 2025, the patient is still alive on continuous combined chemoimmunotherapy; intracranial metastases remain stable without newly developed systemic lesions.

**Figure 1:**
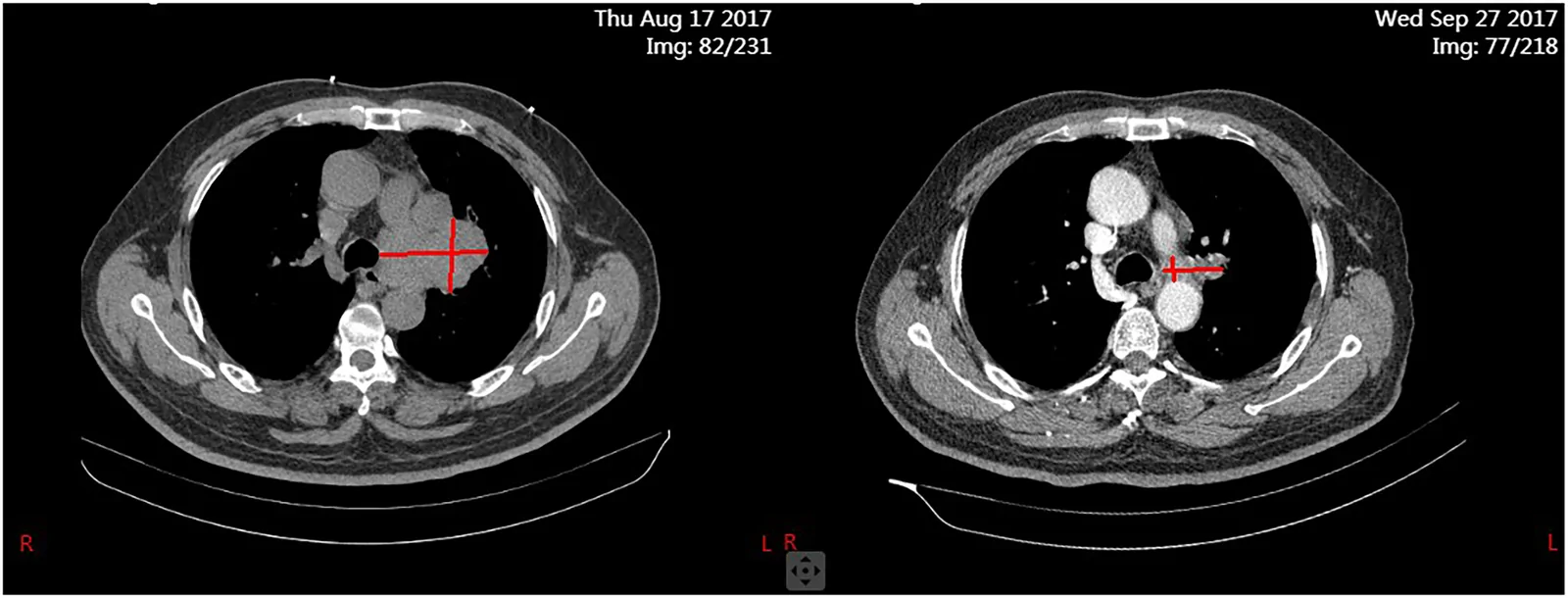
Chest CT cross-sectional scans of Case 1 at the same axial plane before and after chemoradiotherapy for SCLC. All CT scans were performed with a slice thickness of 1.25 mm, and contrast was administered immediately after non-contrast scanning (scanner: United Imaging uCT 960+). The left panel: Pre-treatment typical imaging findings of SCLC: Hilar and mediastinal lymph nodes were enlarged and fused into masses, which severely compressed the superior vena cava into a thin crescent shape (blue hollow circles). This obstruction of venous return in the head, face, and upper limbs led to collateral circulation formation, manifesting as facial and head edema and abnormal dilation of superficial thoracic wall veins. The red calipers indicate tumor measurements, with a cross-sectional area of approximately 74 × 84mm. The right panel: Post chemoradiotherapy, the mass was significantly reduced, and superior vena cava compression was relieved. The cross-sectional area of the tumor (marked by red calipers) is approximately 23 × 34mm.

### Case 2

A 67-year-old male patient presented to our hospital with “chest tightness and hemoptysis for 2 months.” He had a past history of hypertension for 20 years, coronary heart disease, and old inferior myocardial infarction for 2 years. He underwent coronary stent implantation twice, with 2 stents each time, within a month and in two years respectively. Currently, he takes clopidogrel sulfate and aspirin orally to prevent in-stent thrombosis. He had a 10-year smoking history, averaging 20 cigarettes per day, and quit smoking 30 years ago. His family history revealed that his elder brother suffered from gastric adenocarcinoma at age 62, with no other malignant diseases among other first-degree relatives. The patient lived alone. The patient’s ECOG performance status was 1. One month before admission, he developed blood-streaked sputum and underwent a chest X-ray and tumor marker testing at another hospital, which showed an irregular mass-like high-density shadow in the left upper lobe and an elevated neuron-specific enolase (NSE) level (71ng/mL, reference range 1-16.2ng/mL), and the consideration was “to be confirmed the nature of the left upper lung space-occupying lesion”. One week later, he underwent contrast-enhanced chest and abdominal CT, which confirmed left upper lobe lung cancer with regional lymph node metastasis, and a repeat NSE test showed a further elevation to 221 ng/mL. Given the patient’s recent coronary stenting, the treating team fully informed the patient and his family of all available diagnostic options, including fine-needle aspiration biopsy with a clopidogrel washout period. The potential benefits (pathological confirmation) and risks (in-stent thrombosis during washout, bleeding from invasive procedures) were thoroughly discussed. The patient’s cardiologist assessed the risk of in-stent thrombosis during clopidogrel withdrawal as high, given the recent stent implantation and history of old myocardial infarction. After full understanding and careful consideration, the patient and his family voluntarily refused all invasive biopsy procedures. He was admitted to the oncology ward of our hospital for further examination. Admission physical examination was comprehensively performed. Respiratory examination showed equal bilateral thoracic movement, slightly decreased breath sounds in the left hilum region, no wheeze or pleural friction rub; no rales were heard in bilateral lower lungs. Palpation of bilateral cervical and supraclavicular lymph nodes revealed no enlarged, tender or fixed lymph nodes; no palpable masses were detected in the axillary regions. No typical signs of superior vena cava syndrome such as facial edema or dilated superficial thoracic veins were observed. Cardiovascular examination showed normal heart rate and rhythm with no pathological murmurs. The abdomen was soft, non-tender and without hepatosplenomegaly. No obvious cervical venous distension or subcutaneous masses were found, and neurological signs were entirely normal. Four days after admission, whole-body bone imaging and head CT were performed (no distant metastases detected), and NSE was rechecked at 204ng/mL. We re-discussed the biopsy plan with the patient and his family, but they persisted in declining all invasive procedures. Combining the imaging findings and tumor marker results provided by the patient before admission, after a full departmental discussion and Multidisciplinary Team (MDT) consultation, the clinical diagnosis was considered to be small cell lung cancer. No distant metastases were detected by the available imaging examinations, and the disease was tentatively determined to be in the limited stage, requiring timely treatment. Six days after admission, the patient received the first cycle of EC regimen chemotherapy. The specific regimen was: Etoposide injection 100mg intravenous drip on day 1-5 + carboplatin for injection 400mg intravenous drip on day 1. After completing 1 cycle of EC regimen chemotherapy, the patient’s symptoms were significantly relieved. The first chest imaging examination in our hospital was a plain chest CT. The CT showed typical imaging manifestations of small cell lung cancer (left image in **Figure 2**). CT showed multiple soft tissue masses adjacent to the left upper lobe hilum, partially fused into a mass, with the largest level size of approximately 66 × 68mm. Another soft tissue mass was seen under the left apical pleura, with a size of approximately 31 × 64mm. Multiple small nodules were seen adjacent to the left upper lobe pleura, the largest of which was approximately 9 × 10mm. A ground-glass density nodule with a diameter of approximately 3.5mm was seen in the right middle lobe. Multiple strip shadows were seen in the left upper lobe, right middle lobe, and right lower lobe. The mediastinum was not displaced, with enlarged lymph nodes inside. The left hilum was enlarged, with enlarged lymph nodes, and the boundary with the mass was unclear. The heart was not enlarged, and no pleural effusion or pericardial effusion was seen. The right pleura was not thickened. The diagnosis considered central lung cancer in the left upper lobe with multiple metastases in the left lung, left pleural metastasis, and high possibility of left hilar and mediastinal lymph node metastasis. Ground-glass density nodule in the right middle lobe, multiple old lesions in both lungs. Four weeks later, the second cycle of EC chemotherapy was administered, followed by the third cycle four weeks thereafter, with the specific regimen: Etoposide injection 100mg intravenous drip on day 1-5 + carboplatin for injection 400mg intravenous drip on day 1. Two weeks after the third cycle, a follow-up enhanced chest CT showed significant tumor shrinkage (right image in **Figure 2**). Findings of this examination: compared with the old film: the original soft tissue under the left apical pleura basically disappeared, and only a little thickening was seen in the local pleura. The original multiple soft tissue masses adjacent to the left upper lobe hilum mostly disappeared. Now, a strip-like soft tissue shadow is seen in the left hilum, and an oblong soft tissue density shadow is seen in the left upper lobe, with an axial size of approximately 5 × 8mm. Multiple micronodules and ground-glass density nodules are seen in the right lung. Multiple strip shadows are seen in the left upper lobe, right middle lobe, and right lower lobe. The mediastinum was not displaced. The lymph nodes in the aortic window were significantly reduced compared with before, with the largest short diameter of approximately 1.2cm. The left hilum was significantly reduced compared with before, with soft tissue shadows and unclear boundary with the mass. The heart was not enlarged, and no pleural effusion or pericardial effusion was seen. The right pleura was not thickened. The diagnosis considered central lung cancer in the left upper lobe with multiple metastases in the left lung and left pleural metastasis, which was significantly improved compared with before. Multiple micronodules and ground-glass density nodules in the right lung were similar to before. Multiple old lesions in both lungs were the same as before. The efficacy evaluation was partial response (PR). MDT consultation confirmed the patient had limited-stage SCLC with cT3N2M0 (stage IIIB) per AJCC TNM criteria. Given the lack of pathological evidence to confirm SCLC, the MDT withheld the recommendation of PCI. Four weeks after the third cycle, the fourth cycle of EC chemotherapy was administered, and the patient received radiotherapy at another hospital after discharge. The patient was followed up for 27 months with no recurrence. No further follow-up data was available after January 2020, as the patient could not be contacted despite repeated telephone attempts.

**Figure 2:**
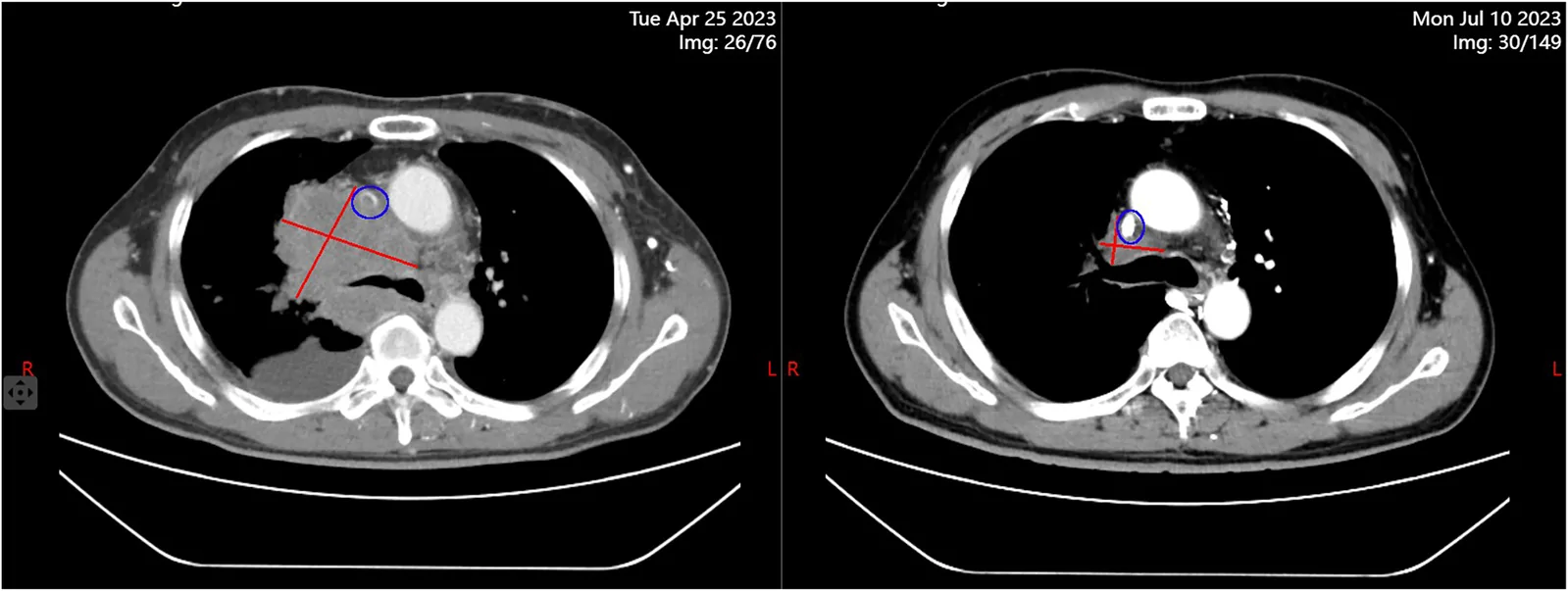
Chest CT cross-sectional scans of Case 2 at the same axial plane before and after first-line EC regimen chemotherapy for limited-stage SCLC. Pre-treatment plain chest CT was performed with a slice thickness of 1.25 mm (scanner: SOMATOM Definition Flash), and post-treatment enhanced CT used the same parameters as Case 1. The left panel: Pre-treatment, enlarged and fused hilar and mediastinal lymph nodes were visible. The red calipers outlines indicate tumor measurements, with a cross-sectional area of approximately 45 × 69mm. The right panel: One cycle after chemotherapy, the mass was significantly reduced. The cross-sectional area of the tumor (marked by red calipers) is approximately 12 × 36mm.

The clinical features and treatment process have been systematically summarized in **Table 1** (Summary of Clinical, Imaging, and Therapeutic Characteristics) and **Table 2** (Timeline), which can provide intuitive references for case detail tracing and diagnosis-treatment logic analysis.

**Table 1:** Clinical, imaging, and therapeutic characteristics of two small cell lung cancer cases.

Characteristic	Case 1	Case 2
Basic Information	56y male	67y male
Medical History	Eczema (2y); 10+y smoking (quit 2y)	Hypertension (20y); CAD + old MI (2y); 2×coronary stenting (2015/2017); 10y smoking (quit 30y)
Clinical Manifestations	Facial/head edema; cough with blood-tinged sputum (2 months); SVCS	Chest tightness; hemoptysis (2 months); blood-streaked sputum
Core Diagnostic Evidence	Pathologically confirmed SCLC; hilar/mediastinal fused lymph nodes (98×74mm); SVC invasion	NSE (221 ng/mL); hilar/mediastinal fused lymph nodes; no pathological sampling
Disease Stage	Extensive-stage SCLC (multiple lymph node metastases; tumor thrombus)	Limited-stage SCLC (no distant metastasis)
TNM staging	cT4N3M1a (stage IVA)	cT3N2M0 (stage IIIB)
Typical CT Imaging Features	Hilar/mediastinal fused lymph nodes; severe SVC compression; tumor thrombus formation	Hilar/mediastinal fused lymph nodes (max 66×68mm); left apical pleural mass (31×64mm)
Core Treatment	EC regimen chemotherapy; proton radiotherapy	EC regimen chemotherapy; sequential radiotherapy
Primary Efficacy Outcome	PR; symptom relief; disease relapse (brain metastasis, 2024)	Clinical CR; tumor disappearance; no recurrence (follow-up to 2020)

CAD, coronary artery disease; CR, complete response; CT, computed tomography; EC, Etoposide+Carboplatin; MI, myocardial infarction; NSE, neuron-specific enolase; PR, partial response; SCLC, small cell lung cancer; SVCS, superior vena cava syndrome; TNM, tumor, node, metastasis; y, year.

**Table 2:** Clinical timeline of two small cell lung cancer cases.

Time Point	Case 1	Case 2
2023 Feb	Onset of SVCS symptoms; pathological confirmation of SCLC (bronchoscopic biopsy)	–
2023 Apr	Admission; initiation of EC chemotherapy; PET-CT confirms extensive-stage	–
2023 Jul	Post-chemoradiotherapy CT: tumor regression (PR); SVCS symptom relief	–
2024 Mar	Brain metastasis detected; cranial radiotherapy	–
2025 May	Restarted chemotherapy + immunotherapy (ongoing treatment)	–
2017 Jul	–	Onset of hemoptysis; elevated NSE (71 ng/mL); chest X-ray shows lung mass
2017 Aug	–	Admission; MDT clinical diagnosis of limited-stage SCLC; initiation of EC chemotherapy
2017 Sep	–	Post-chemotherapy CT: tumor regression (PR); continued EC chemotherapy
2017 Oct	–	Completion of 4 cycles EC chemotherapy; sequential radiotherapy
Post-2017 Oct	–	Achieved clinical CR; tumor disappearance
Follow-Up End	July 2025 (ongoing treatment, relapsed)	January 2020 (no recurrence, clinical CR maintained). The patient remained disease-free for roughly 27 months; follow-up was discontinued in January 2020 due to loss to follow-up.

CR, complete response; EC, Etoposide+Carboplatin; MDT, Multidisciplinary Team; NSE, neuron-specific enolase; PET-CT, positron emission tomography-computed tomography; PR, partial response; SCLC, small cell lung cancer; SVCS, superior vena cava syndrome.

## Discussion

Chest CT is the principal non-invasive tool for evaluating SCLC. Its characteristic findings correlate closely with the tumor’s biology and with clinical symptoms, making CT central to early recognition. Both patients exhibited the classic imaging pattern of coalescent hilar and mediastinal lymphadenopathy. SCLC arises from bronchial neuroendocrine cells, grows invasively, and metastasizes early to regional lymph nodes; this behavior directly underlies the coalescent nodal pattern on CT. Unlike the scattered mediastinal lymphadenopathy typical of non-small cell lung cancer (NSCLC), coalescent nodal masses have important differential diagnostic value and provide a key clue for suspecting SCLC (14).

Case 1 illustrates this typical CT pattern. The accompanying superior vena cava (SVC) syndrome and venous tumor thrombi highlight why recognizing these features matters: they not only support the diagnosis but also reveal life-threatening complications that require urgent treatment. The two cases are complementary. Case 1 validates the specificity of the typical imaging pattern, whereas Case 2 demonstrates its practical application when pathology is unobtainable.

The correlation between imaging findings and clinical symptoms further enhances the credibility of diagnosis. In Case 1, contrast-enhanced CT demonstrated severe SVC compression, with the SVC lumen narrowed to a thin crescent shape; this finding was closely correlated with the patient’s clinical manifestations of facial and cephalic edema and dilated superficial thoracic wall veins. This imaging-clinical correlation provided a mechanistic explanation for how coalescent SCLC lymph node masses compress adjacent thoracic structures and induce severe complications (15). Imaging findings provided evidence for the diagnosis of superior vena cava syndrome, prompting the timely initiation of chemotherapy (14). Current guidelines still uphold the principle that pathological examination is the “gold standard” for SCLC diagnosis (12). However, in certain clinical scenarios, pathological confirmation may be unfeasible due to inherent constraints. Case 2 had recently undergone coronary artery stenting and needed to take antiplatelet drugs continuously, resulting in a significantly increased risk of bleeding during biopsy. In this case, the combined diagnostic model of “typical imaging features + specific tumor markers” was adopted, which not only avoided the risk of invasive procedures but also achieved timely diagnosis. In Case 2, the level of NSE was as high as 221 ng/mL, significantly exceeding the normal reference range (16). As a specific tumor marker for SCLC, the abnormal increase in NSE and the imaging feature of “hilar and mediastinal fused lymph nodes” formed “dual evidence,” improving the accuracy of diagnosis and preventing delays in treatment due to waiting for pathological results.

Although the combination of typical SCLC imaging features (hilar/mediastinal fused lymph nodes) and markedly elevated NSE provided strong circumstantial evidence for clinical diagnosis in Case 2, it is imperative to emphasize that neither indicator is specific to SCLC. NSE can be elevated in other neuroendocrine tumors, small round blue cell tumors, and even non-malignant conditions (e.g., brain injury, inflammatory diseases) (16); fused mediastinal masses may also occur in sarcoidosis, lymphoma, or metastatic small round cell tumors. Thus, the “imaging + NSE” model is never intended to replace pathological confirmation. Instead, it serves as a tentative diagnostic tool reserved for scenarios where pathology is unfeasible despite maximal efforts, and its application requires strict differential diagnosis and MDT consultation to minimize misdiagnosis risk. Lymphoma and NSCLC are the primary differential diagnoses due to their clinical and imaging overlap with SCLC. Lymphoma typically manifests as symmetric, well-circumscribed coalescent lymphadenopathy in the anterior/middle mediastinum with uniform enhancement on enhanced CT, in contrast to SCLC’s invasive hilar/peribronchial fused lymph nodes that often compress adjacent structures (e.g., superior vena cava); lymphoma also spares the pulmonary parenchyma, does not cause bronchial obstruction, is unassociated with marked NSE elevation, and typically presents with systemic symptoms (fever, night sweats, weight loss) absent in our case. NSCLC is distinguished by scattered, non-fused mediastinal lymph node enlargement (short diameter < 2 cm), a predominantly peripheral origin, and association with elevated CEA/CYFRA21-1 (rather than NSE); normal CEA/CYFRA21–1 in our case supported its exclusion. Other mimics were easily ruled out by core features: low-grade neuroendocrine tumors (NETs) have small, well-defined masses and mild NSE elevation; tuberculous lymphadenitis shows calcification/caseous necrosis and inflammatory marker elevation; sarcoidosis presents with bilateral symmetric hilar lymphadenopathy, interstitial lung changes and elevated ACE; small round blue cell tumors rarely involve the lung as a primary site and do not respond to SCLC-standard chemoradiotherapy—none of these features were present in our case.

## Ethical considerations

The decision to initiate chemotherapy without histological confirmation was guided by the four principles of biomedical ethics. Respect for autonomy was maintained through full disclosure of the diagnostic uncertainty and alternative options; the patient’s voluntary refusal of invasive biopsy did not preclude access to treatment. Non-maleficence required balancing the risks of biopsy (bleeding, stent thrombosis) against the risks of treatment delay; both the cardiologist’s assessment and the MDT consensus favored avoiding procedural harm. Beneficence supported timely intervention given the tumor’s rapid doubling time and the high probability of response, with early reassessment providing a safety net. Justice demanded that the patient not be denied standard care due to an inability to tolerate diagnostic procedures. This framework supports the use of clinical diagnosis combined with MDT oversight as an ethically sound approach when tissue confirmation is contraindicated. Written informed consent was signed by the patient and his family, with explicit documentation acknowledging the absence of histological confirmation and voluntary acceptance of empirical anti-tumor therapy.

## Limitation

This study has the following limitations. First, the staging evaluation for Case 2 relied on head CT rather than brain MRI, which is the recommended standard for SCLC staging. Brain MRI has superior sensitivity for detecting small brain metastases, and the use of head CT may have led to potential understaging. This limitation highlights the importance of adhering to current staging guidelines to ensure accurate assessment of distant metastasis. Second, the study did not incorporate liquid biopsy or circulating tumor DNA (ctDNA) testing, which are now standard-of-care options for SCLC diagnosis in 2025 when tissue pathology is unobtainable (17–19). At the time of Case 2’s diagnosis (2017), these technologies were not widely available in clinical settings. However, in current practice, ctDNA testing offers higher specificity for SCLC than NSE and can effectively differentiate SCLC from overlapping diseases, which would have enhanced the diagnostic rigor of the pathology-unobtainable scenario. This limitation underscores the importance of combining traditional imaging/tumor markers with modern molecular diagnostic tools to optimize diagnostic strategies for future similar cases. Third, we only tested NSE without detecting progastrin-releasing peptide (ProGRP) in Case 2. ProGRP exhibits better diagnostic efficacy for SCLC than NSE, with higher diagnostic sensitivity especially for limited-stage SCLC (20). Unfortunately, the clinical laboratory of our hospital had not yet launched the ProGRP detection assay in 2017 when the patient received treatment, so relevant ProGRP data could not be collected for auxiliary diagnosis. This missing ProGRP test result represents another important limitation of the present study. Fourth, in Case 2, the diagnostic probabilities and risk–benefit weighing were documented qualitatively in 2017; no formal quantitative risk–benefit analysis was performed at the time. Prospective use of structured decision frameworks — explicit pre-treatment probability estimation, predefined response checkpoints, and standardized documentation templates — would strengthen the rigor of managing “pathology-unobtainable” scenarios. In addition, Case 2 achieved clinical CR, which is relatively rare in SCLC given its well-recognized poor prognosis. This particular clinical outcome may not be representative of the broader SCLC population. Finally, although the ethical principles outlined above guided the clinical decision-making, no formal ethics committee consultation was sought at the time, as the management was considered to fall within standard clinical practice for MDT decision-making. Prospective documentation of the ethical reasoning process would strengthen similar future cases.

## The patients’ perspectives

Patient 1: I first went to the hospital because of facial swelling, but was diagnosed with lung cancer—and it was quite advanced. Learning that it’s a tumor with a poor prognosis, I was firmly against radiotherapy and chemotherapy at first. Later, I drank traditional Chinese medicine tea for a period of time, and the facial swelling improved. I was torn about whether to give treatment a try. After completing comprehensive examinations at a Grade A tertiary hospital in Qingdao, I was admitted to a hospital in Beijing for treatment. At one point, my condition improved significantly, and all aspects of my health got much better—I truly felt that deciding to undergo treatment was the right choice. Later, the disease relapsed, but I am still receiving treatment. Since my diagnosis, my family has been extremely supportive. I am deeply grateful to my family, as well as the doctors and nurses I have encountered—they have all been very dedicated and responsible.

Patient 2: Unfortunately, we cannot collect self-reported experience from the second patient. The last regular follow-up ended in January 2020. Subsequent telephone follow-up attempts failed to reach the patient, and no subsequent medical visit records for this individual could be retrieved from our system.

## Conclusions

In clinical settings where pathological sampling is not achievable despite all reasonable attempts, a comprehensive clinical assessment incorporating typical SCLC imaging findings, marked elevation of tumor markers, and characteristic clinical manifestations may be used as a pragmatic last-resort strategy. Importantly, this approach is not intended to replace pathological examination, which remains the undisputed gold standard for SCLC diagnosis. Clinicians must prioritize pathological sampling whenever possible and strictly rule out alternative diagnoses before initiating treatment based on non-pathological evidence. When pathological results cannot be obtained, the alternative diagnostic methods and their efficacy still require further exploration and verification through future research.

## Data Availability

The raw data supporting the conclusions of this article will be made available by the authors, without undue reservation.
